# Supplementary material for: Mitochondrial translation deficiency impairs NAD+‐mediated lysosomal acidification
Source: EMBO J. 2021 Feb 2;40(8):e105268. doi: 10.15252/embj.2020105268 (PMC8047443; doi:10.15252/embj.2020105268)
Supplement: Supplementary file 2 — Expanded View Figures PDF [file EMBJ-40-e105268-s004.pdf]

Expanded View Figures

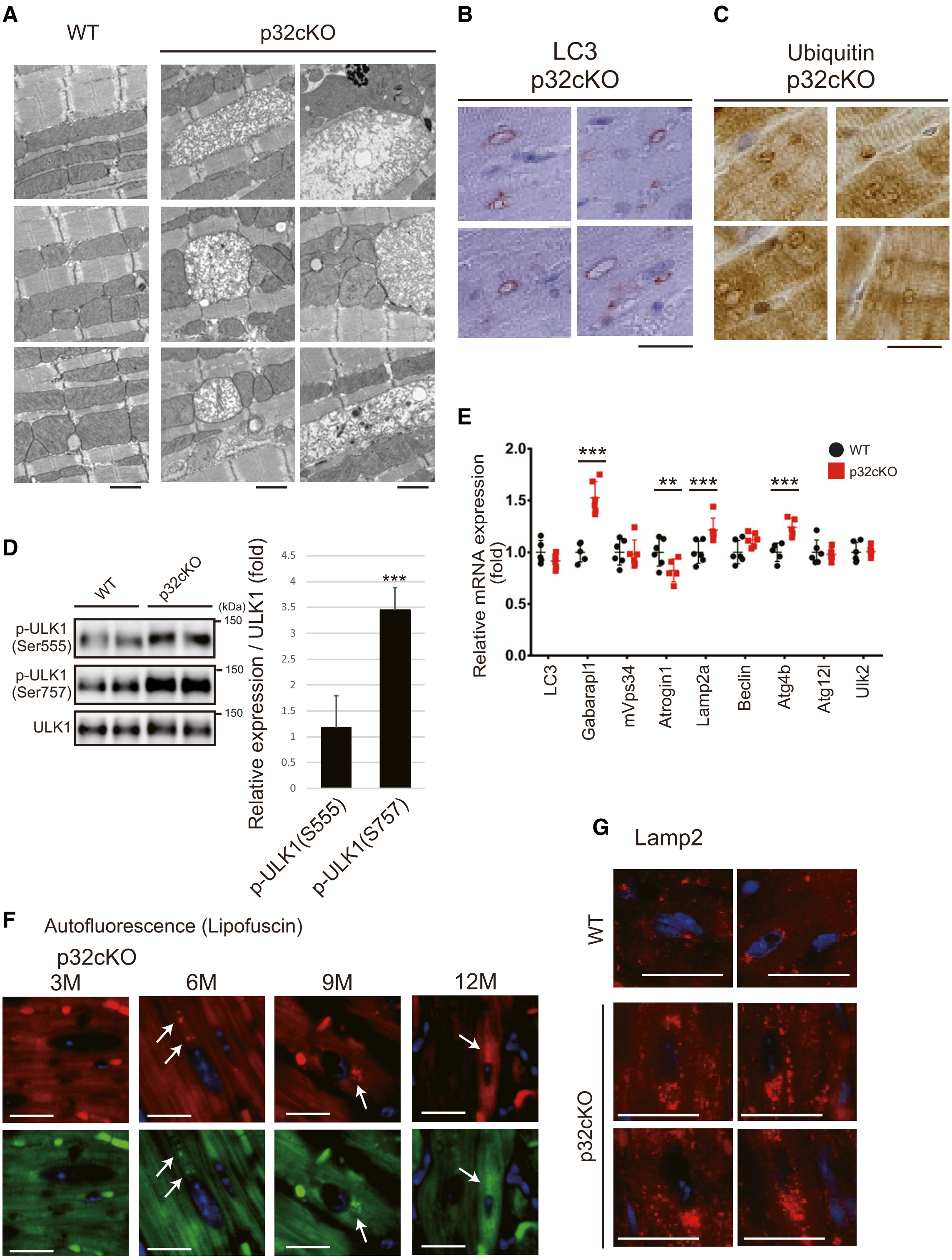

Figure EV1.

 © 2021 The Authors

◀ **Figure EV1. Autophagy and lysosomal function are suppressed in the heart of p32cKO mice.**

- A Electron microscopy of hearts from 6-month-old mice ($n = 6$). EMs of three WT and six p32cKO heart were presented. Two of them are posted as used in Fig 1A. p32cKO hearts showed morphologically abnormal mitochondria. Scale bars, 1 μm.
- B, C Immunostaining of LC3 (B) and ubiquitin (C) in the 6-month-old p32cKO heart tissues. Only p32cKO hearts showed ring-shaped structures ($n = 4$). Scale bar, 20 μm.
- D Western blot analysis of phosphorylated ULK1. The p-ULK1(Ser757) levels were higher in the 9-month-old p32cKO hearts than in WT hearts ($n = 6$ mice per group). The p-ULK1(Ser555) levels were similar in p32cKO and WT hearts. Relative expression of phosphorylated ULK1 is shown on the right. Error bars are presented as mean ± SD. Statistical significance was assessed by Student's *t*-test, $^{***}P < 0.005$.
- E Real-time PCR analysis of RNA expression of autophagy-related genes in the 6-month-old hearts of WT and p32cKO mice ($n = 6$). Error bars are presented as mean ± SD. Statistical significance was assessed by Student's *t*-test, $^{**}P < 0.01$, $^{***}P < 0.001$.
- F Autofluorescence showing lipofuscin localization around the nucleus in the p32cKO heart at different ages. Tissues were excited at a wavelength of 540 (upper panel) or 470 (lower panel) and emission spectra were collected with a confocal microscope at wavelengths (band path) of 580–630 nm (upper panel) or 510–560 nm (lower panel). Lipofuscin is indicated by arrows. Scale bars, 20 μm.
- G Immunostaining of Lamp2 in the heart of 12-month-old mice using TrueBlack™ to quench lipofuscin autofluorescence. In the p32cKO heart, but not in WT, many dots around nuclei were observed. Scale bars, 20 μm.

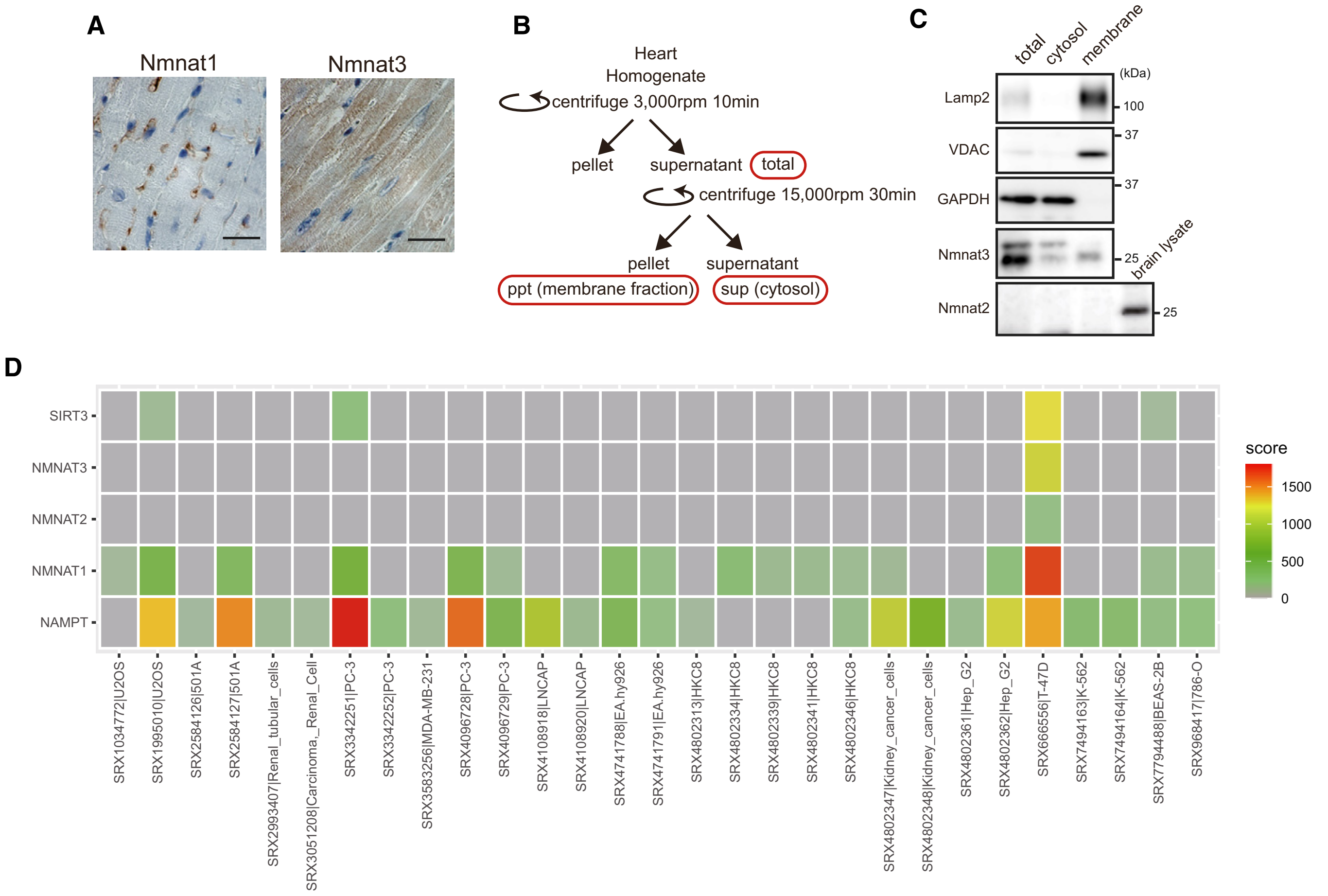

Figure EV2. Expression and localization of Nmnat3 and regulation by HIF1α.

- A Immunostaining of Nmnat1 (left) and Nmnat3 (right) in the 9-month-old WT heart. Nmnat1 and Nmnat3 are localized in the nucleus and cytosol, respectively. Scale bar, 20 μm. One representative experiment out of three shown.
- B A schema of the procedure of membrane fraction and cytosol purification.
- C Western blot analysis of Nmnat3 in the 3-month-old WT mouse heart. Nmnat3 expression was observed in both cytosolic and membrane fractions. Lamp2 and VDAC are membrane fraction markers, and PGK1 and GAPDH are cytosolic markers. The expression of Nmnat2 was not observed in heart, but expressed in the 2-month-old WT mouse brain. One representative experiment out of three shown.
- D HIF1α was associated with the promoter region of *Nampt*, *Nmnat1–3*, and *Sirt3* in several cell lines. We used the public database, ChIP-Atlas (http://chip-atlas.org/).

© 2021 The Authors

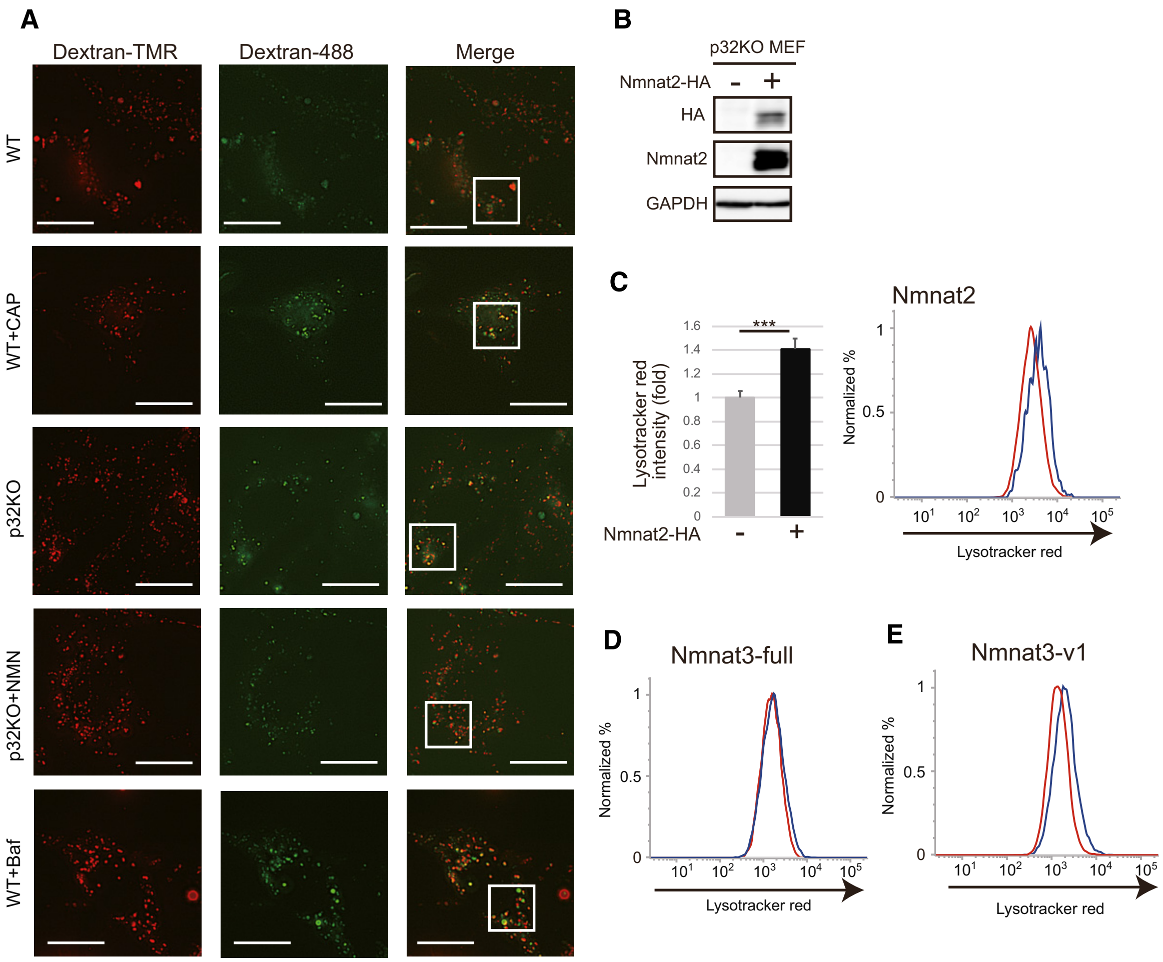

Figure EV3. Lysosomal function was rescued by Nmnat2 and Nmnat3 overexpression.

A Lysosomal acidification was impaired by treatment with CAP and in p32KO MEFs. Representative images of WT and p32KO MEFs stained with dextran-Oregon Green (488) and dextran-TMRM. Scale bar, 5 μm. Addition of 1 mM NMN for 48 h to p32KO MEFs rescued lysosomal acidification, while 1 mM CAP treatment of WT MEFs for 48 h decreased lysosomal acidification. Enlarged view of the white squares is shown Fig 4A. One representative experiment out of three shown.

B Nmnat2-HA was transfected into p32KO MEFs. Western blot analysis of anti-HA and anti-Nmnat2 is shown. GAPDH was used as a loading control.

C Overexpression of Nmnat2-HA cells into p32KO MEFs increased LysoTracker Red staining. The light side was analyzed by FACS with LysoTracker Red. Error bars are presented as mean ± SEM of three independent experiments. Statistical significance was assessed by Student's *t*-test, ***$P < 0.001$.

D, E FACS analysis was performed with LysoTracker Red on p32KO MEFs overexpressing Nmnat3(full) (D) or Nmnat3(v1) (E). One representative experiment out of three shown.

 © 2021 The Authors

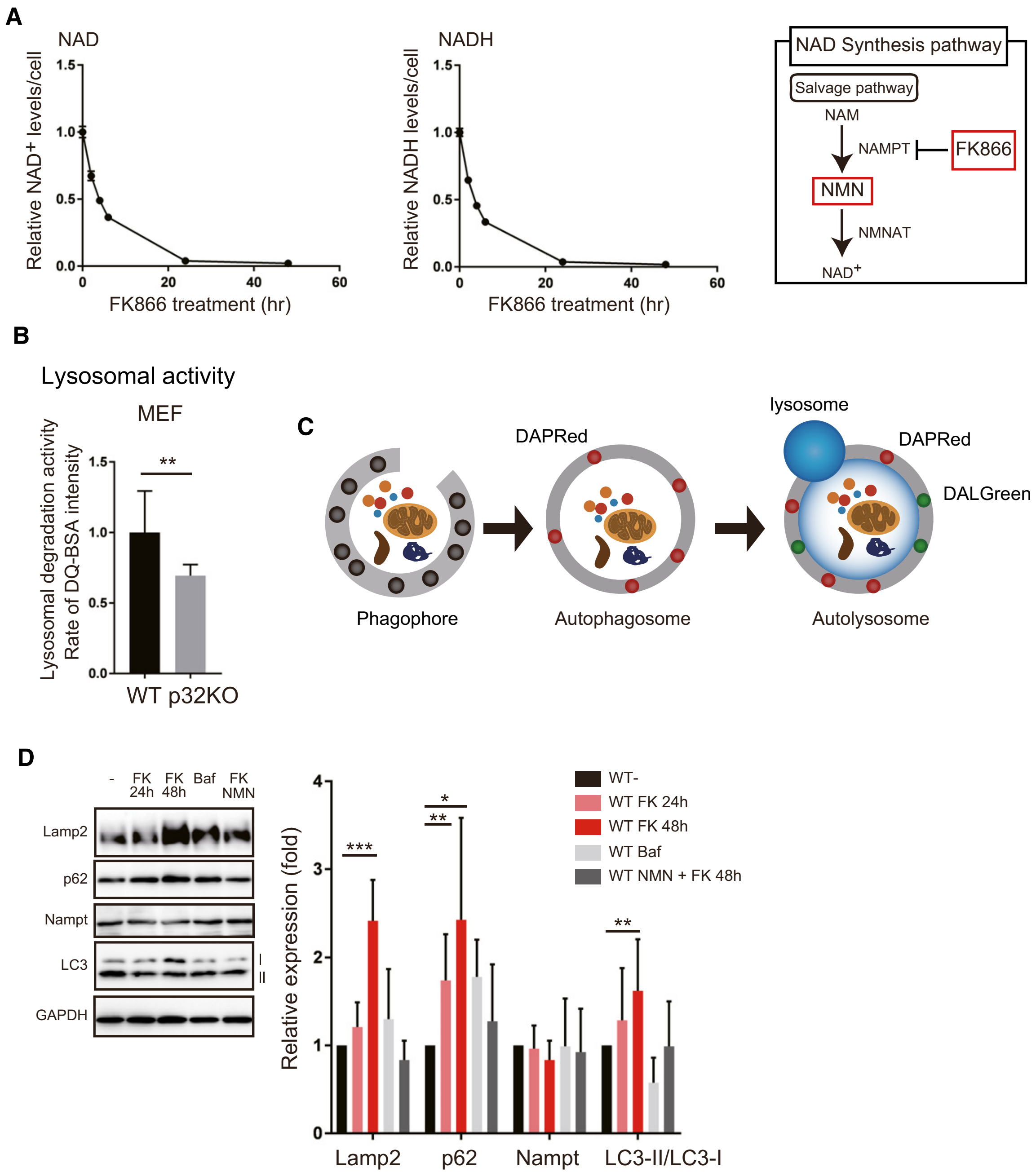

Figure EV4. The Nampt inhibitor, FK866, decreased NAD+ contents and lysosomal function.

A Time course of intracellular NAD+ or NADH content after addition of the Nampt inhibitor, FK866 ($n = 3$) in WT MEF cells. The right panel shows what FK866 inhibits in the NAD synthesis pathway.

B Lysosomal activity was measured by DQ-BSA in WT and p32KO MEFs. Error bars are presented as mean ± SEM of three independent experiments. Statistical significance was assessed by Student's *t*-test, $^{**}P < 0.002$.

C To monitor autophagosomes and autolysosomes, DAPRed and DALGreen were used. DALGreen fluorescence is enhanced at an acidic pH and is suitable for monitoring the autophagy degradation stage, also known as the autolysosome stage. In contrast, DAPRed has a pH-independent fluorescence profile and remains fluorescent with almost constant intensity throughout the process of autophagy.

D Western blot analysis of Lamp2, p62, Nampt, and LC3. Treatment of WT MEFs with FK866 increased the expression of Lamp2, p62, and LC3. When bafilomycin A or FK866 plus NMN were added, the expression of these proteins did not change. One representative experiment out of three shown. Quantification is shown on the right side. The values are mean ± SD of three independent experiments. Statistical significance was assessed by Student's *t*-test, $^{*}P < 0.05$, $^{**}P < 0.01$, $^{***}P < 0.005$ ($n = 4$ per group).

© 2021 The Authors

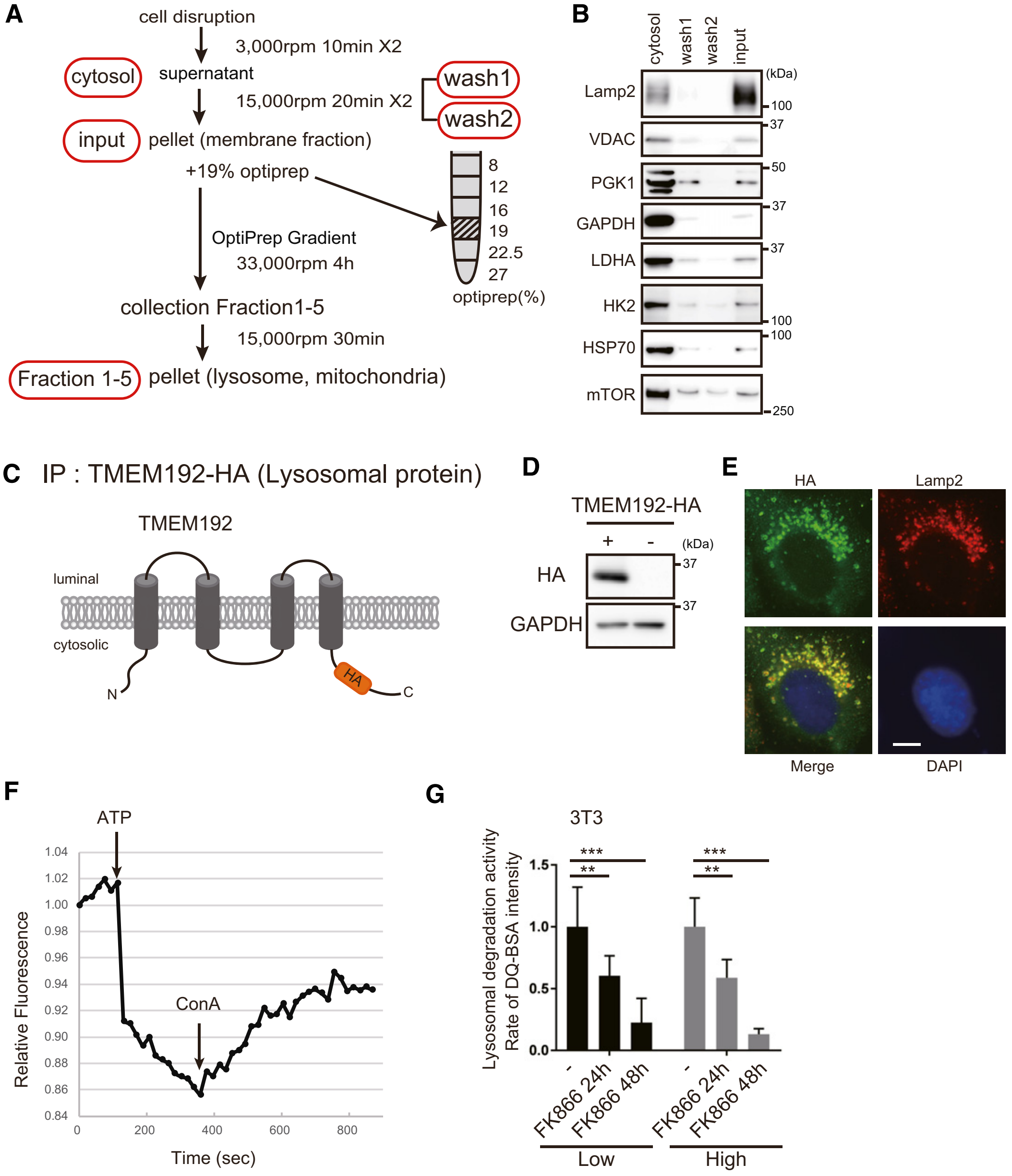

Figure EV5. Purification and function analysis of lysosomes.

A Schema of lysosome purification by centrifugation. The samples were washed twice to reduce cytosolic protein contamination.

B The lysosomal fractions in WT MEFs were subjected to Western blotting to examine the presence of characteristic organelle marker proteins. After two washes and centrifugations, the membrane fraction was concentrated, and very little protein was detected, suggesting that this method results in little cytosolic protein contamination.

C Schema of the HA-tagged construct of lysosomal protein TMEM192.

D, E The expression of TMEM192 in 3T3-L1 cells was examined by Western blotting and immunostaining (HA: Green and Lamp2: Red). Scale bar, 10 μm.

F Lysosomal fraction isolated with MAG10 was in the active state in 3T3-L1 cells. V-ATPase activity was monitored by ACMA, which is a fluorescent pH indicator. The activity was started using ATP and inhibited after addition of concanavalin A (ConA). One representative experiment out of three shown.

G Lysosomal activity was measured by DQ-BSA. Two types of culture medium were used: low- and high-glucose DMEM. Decreased lysosomal proteolytic capacity was observed after FK866 treatment of 3T3-L1 cells. However, the glucose concentration had no effect. Error bars are presented as mean ± SEM of three independent experiments. Statistical significance was assessed by one-way ANOVA, $^{**}P < 0.005$, $^{***}P < 0.001$.

© 2021 The Authors
