# Supplementary figures and images for: Mitochondrial translation deficiency impairs NAD+‐mediated lysosomal acidification
Source: EMBO J. 2021 Feb 2;40(8):e105268. doi: 10.15252/embj.2020105268 (PMC8047443; doi:10.15252/embj.2020105268)

Figure 1

B

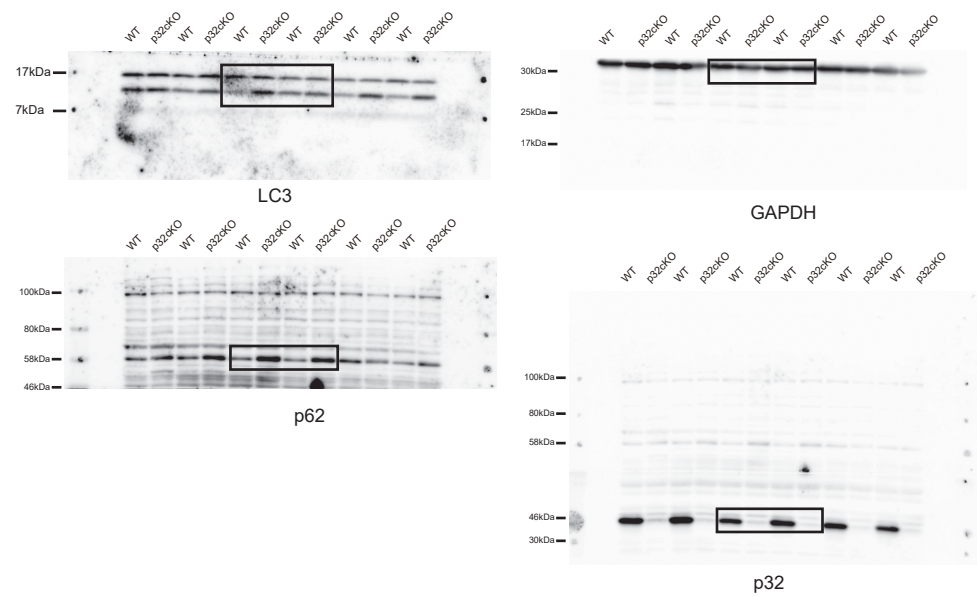

F

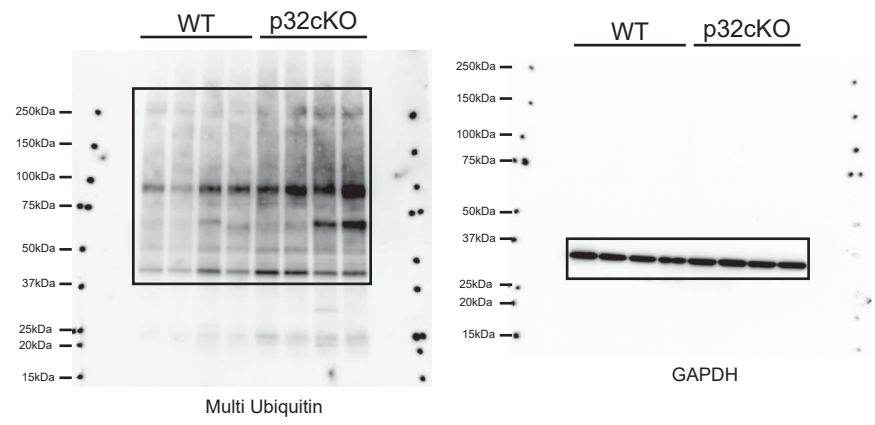

G

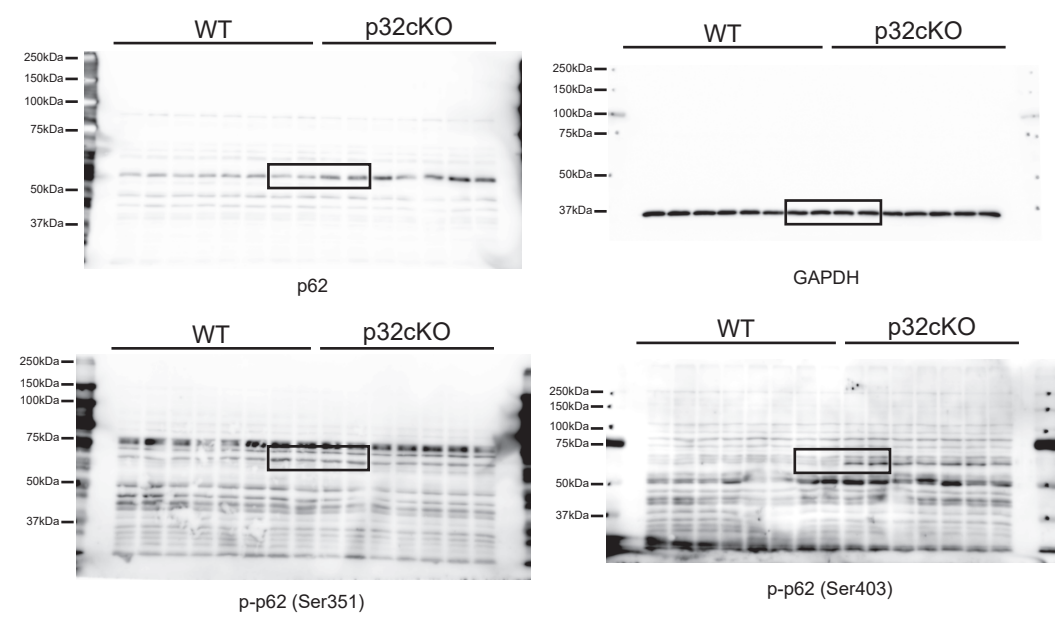

Supplement: Supplementary file 3 — Source Data for Figure 1 [file EMBJ-40-e105268-s006.pdf]

Figure 2  
D

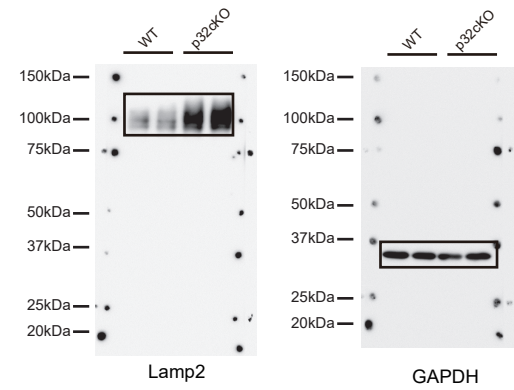

Supplement: Supplementary file 4 — Source Data for Figure 2 [file EMBJ-40-e105268-s005.pdf]

Figure 3

C

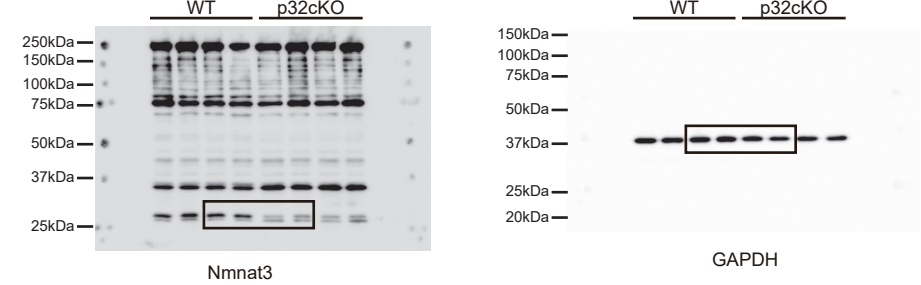

E

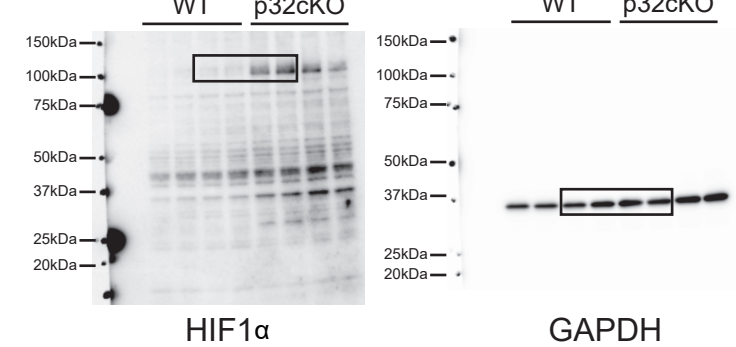

F

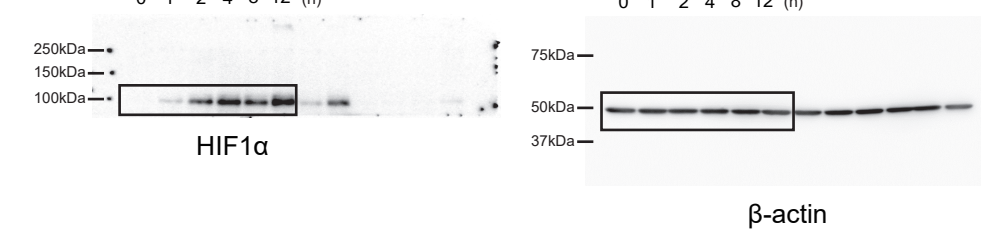

H

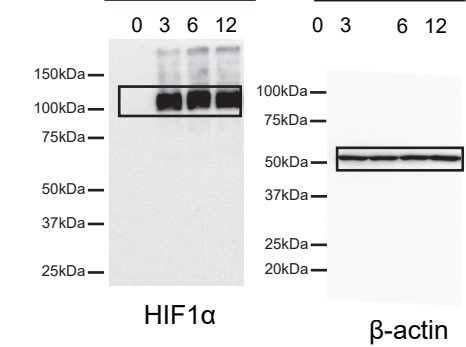

Supplement: Supplementary file 5 — Source Data for Figure 3 [file EMBJ-40-e105268-s001.pdf]

Figure 4

C

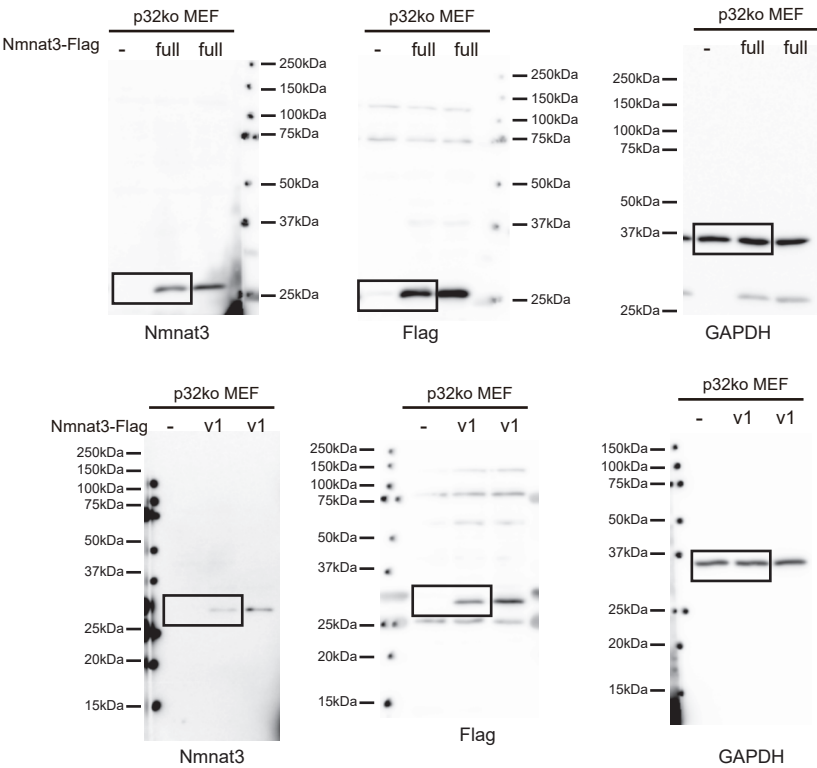

Supplement: Supplementary file 6 — Source Data for Figure 4 [file EMBJ-40-e105268-s002.pdf]

Figure 6

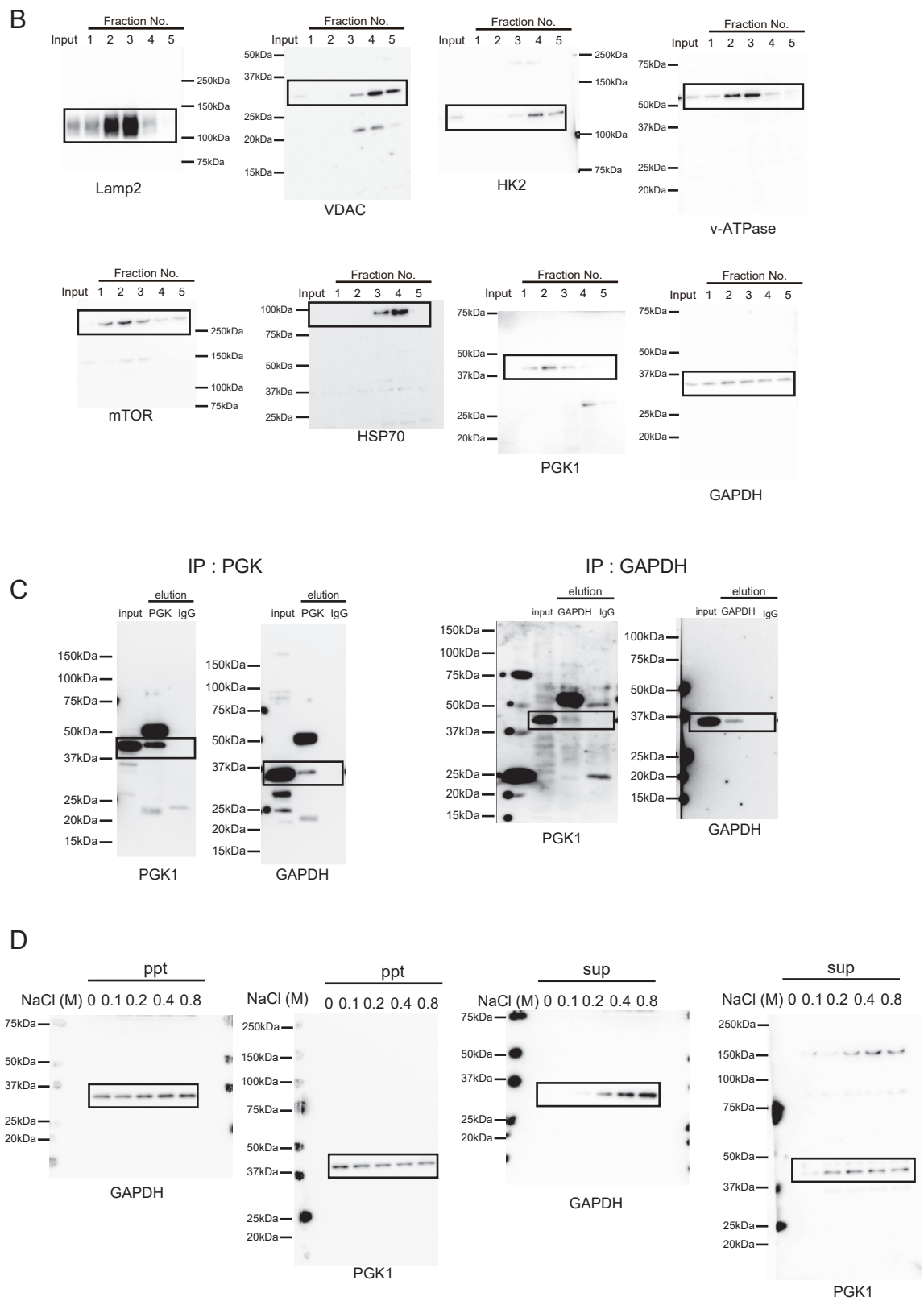

Figure 6

G

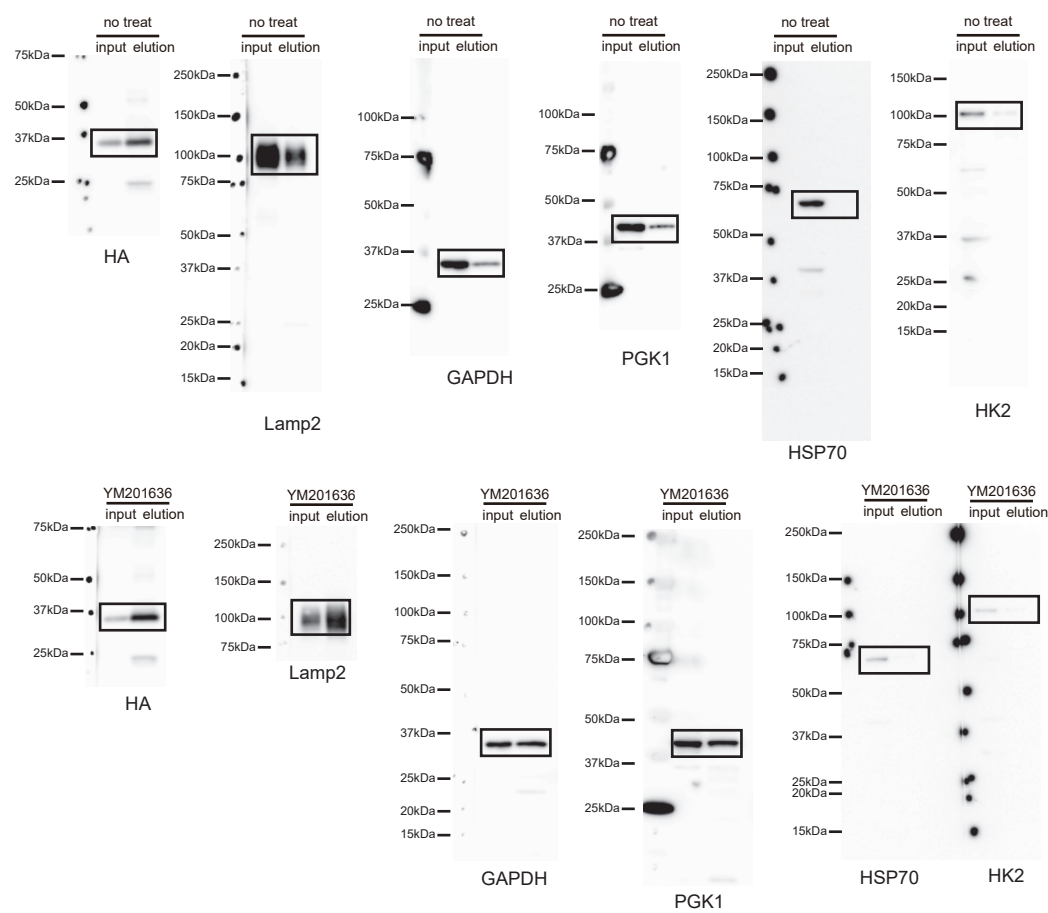

H

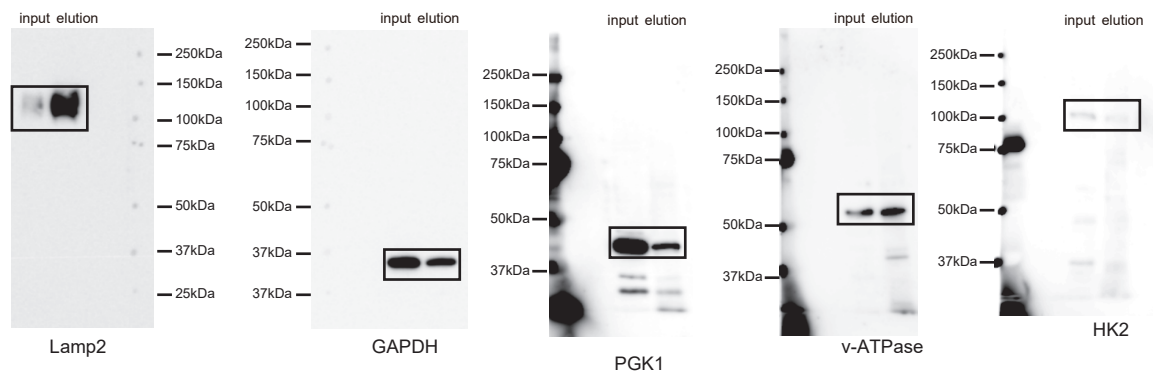

Supplement: Supplementary file 7 — Source Data for Figure 6 [file EMBJ-40-e105268-s007.pdf]
